# How exogenous nitric oxide regulates nitrogen assimilation in wheat seedlings under different nitrogen sources and levels

**DOI:** 10.1371/journal.pone.0190269

**Published:** 2018-01-10

**Authors:** Sadegh Balotf, Shahidul Islam, Gholamreza Kavoosi, Bahman Kholdebarin, Angela Juhasz, Wujun Ma

**Affiliations:** 1 School of Veterinary and Life Science, Murdoch University, Perth, Western Australia, Australia; 2 Institute of Biotechnology, Shiraz University, Shiraz, Iran; 3 Department of Biology, Faculty of Sciences, Shiraz University, Shiraz, Iran; Institute of Genetics and Developmental Biology Chinese Academy of Sciences, CHINA

## Abstract

Nitrogen (N) is one of the most important nutrients for plants and nitric oxide (NO) as a signaling plant growth regulator involved in nitrogen assimilation. Understanding the influence of exogenous NO on nitrogen metabolism at the gene expression and enzyme activity levels under different sources of nitrogen is vitally important for increasing nitrogen use efficiency (NUE). This study investigated the expression of key genes and enzymes in relation to nitrogen assimilation in two Australian wheat cultivars, a popular high NUE cv. Spitfire and a normal NUE cv. Westonia, under different combinations of nitrogen and sodium nitroprusside (SNP) as the NO donor. Application of NO increased the gene expressions and activities of nitrogen assimilation pathway enzymes in both cultivars at low levels of nitrogen. At high nitrogen supplies, the expressions and activities of N assimilation genes increased in response to exogenous NO only in cv. Spitfire but not in cv. Westonia. Exogenous NO caused an increase in leaf NO content at low N supplies in both cultivars, while under high nitrogen treatments, cv. Spitfire showed an increase under ammonium nitrate (NH_4_NO_3_) treatment but cv. Westonia was not affected. N assimilation gene expression and enzyme activity showed a clear relationship between exogenous NO, N concentration and N forms in primary plant nitrogen assimilation. Results reveal the possible role of NO and different nitrogen sources on nitrogen assimilation in *Triticum aestivum* plants.

## Introduction

Nitrogen (N) is not only one of the essential macro-nutrients for plants but also a major limiting mineral element for plant growth and yield [1]. Improving the N use efficiency (NUE) in crops is fundamental for modern agriculture. The production of nitrogen fertilizers is expensive and time consuming, thus high NUE is essential for agriculture productivity. Meanwhile, excessive use of nitrogen will cause environmental pollution [2, 3]. For wheat and other grain crops, NUE is traditionally defined as the grain yield per unit of available N in the soil and is composed of two processes i.e. uptake efficiency and the utilization efficiency [4–6]. A number of physiological and environmental traits can affect the NUE in plants, including N source [7, 8], N concentration and the remobilization of N from senescent tissues [2].

Ammonium (NH_4_^+^) and nitrate (NO_3_^−^) are two major nitrogen sources for most plant species. The form of nitrogen available to plants can affect leaf expansion and function, gene expression pattern, root architecture, and partitioning of dry matter between leaves and roots [9–11]. With the exception of NH_4_^+^-tolerant species, most plants prefer NO_3_^−^ over NH_4_^+^ as the primary nitrogen source even though more energy is needed for NO_3_^−^ assimilation [12, 13]. Using NH_4_^+^ as the sole N source may be toxic to plants after being combined with the internal NH_4_^+^ produced through metabolic processes. However, studies have shown that the toxic effects of external NH_4_^+^ supply can be relieved by applying ammonium together with nitrate as NH_4_NO_3_ [14].

The processes of NO_3_^-^ uptake by the roots and its assimilation in the leaves involve NO_3_^-^ transportation into the leaf cells and then transportation of nitrite (NO_2_^-^), the product of nitrate reduction, in cytoplasm into the chloroplasts. A two steps reduction catalyzed by nitrate reductase (NR; EC 1.7.1.1/2) and nitrite reductase (NiR; EC 1.7.7.1) leads to the formation of NH_4_^+^ as the final product. Ammonium ion is assimilated into glutamine and then into glutamate by the activities of glutamine synthetase (GS; EC 6.3.1.2) and ferredoxin/NADH-glutamate synthase (Fd-GOGAT; EC 1.4.7.1 or NADH-GOGAT; EC 1.4.1.14) enzymes [15]. Two GS isoforms exist in the genome of higher plants, the cytosolic glutamine synthetase (GS1) and the chloroplastic glutamine synthetase (GS2). The cytosolic isoform is important for assimilating NH_4_^+^ for both primary N assimilation and its recycling, while plastidic GS2 and Fd-GOGAT enzymes have been reported to be the crucial ones for the re-assimilation of photorespiratory produced NH_4_^+^ [16, 17]. Recent reports also have highlighted a role of nitric oxide (NO) in N assimilation and N uptake in plants and have suggested that NO is an important signaling molecule of the nitrate-sensing pathway [18, 19]. Nitric Oxide is a small redox signal molecule in plant cells that acts as a signaling plant growth regulator. There are several possible processes leading to NO formation in plants, enzymatically or non-enzymatically from nitrite or from arginine by NO synthase (NOS) as in animals [20]. NR and NOS are two main enzymes involved in NO production in plants, although the presence of NOS in plants is still questionable [21]. Despite the importance of NO in plant signaling, little is known about the mechanisms of NO-regulated N assimilation in response to different nitrogen sources.

Wheat (*Triticum aestivum* L.), one of the most important strategic food crops globally, is cultivated worldwide. Understanding the molecular responses of wheat to nitrogen and nitric oxide supplies and their relationships to physiological indicators may provide a reliable solution to increase NUE in this crop. In the present study, the relationships between the transcript levels of six key nitrogen assimilation genes (TaNR, TaNiR, TaGS1, TaGS2, TaFd-GOGAT and TaNADH-GOGAT) and wheat seedlings’ physiological performance under different combinations of nitrogen and sodium nitroprusside (SNP) has been examined in two Australian wheat cultivars with contrasting NUE.

## Material and methods

### Plant growth conditions and nitrogen treatment

Two Australian wheat cultivars (cvs Westonia and Spitfire) were used in the present study. Their NUEs differ significantly. Spitfire is known as a high protein content or NUE cultivar and Westonia is ranked medium in respect of both protein content and grain yield. Wheat seedlings were grown in nutrient solution similar to Hoagland solution in glasshouse under natural light conditions for two weeks. 3 kg pots with 5 seedlings per pot were used in this excrement. After this period, plants were irrigated with nitrogen-free nutrient solution for a week. Plants treated with different chemical forms of nitrogen with 0, 20 or 100 μM of SNP, were irrigated with nutrient solution containing 4 mM /40 mM KNO_3_ (NO_3_^−^ plants), 4 mM /40 mM NH_4_Cl (NH_4_^+^ plants), or 4 mM /40 mM NH_4_NO_3_ (NH_4_NO_3_ plants) for 3 days. Plants were supplied with different combination of N and NO at the rate of 50 ml per pot daily, which was adopted from *Balotf* et al. [22]. To prevent ammonium oxidation by nitrifying bacteria, 8.0 μM dicyandiamide, a nitrification inhibitor, was added to each pot. Leaf tissues were harvested 24 hours after nitrogen treatments. Every harvest consisted of three independent biological replicates for each genotype and treatment.

### RNA extraction and qRT–PCR

Leaves materials were frozen in liquid nitrogen, homogenized in a mortar and pestle, and kept at −80°C until used. Total RNA was isolated using RNeasy Plant Mini Kit (Qiagen) following the manufacturer’s protocol. For qRT–PCR analysis, total RNA was treated with the DNase I (Qiagen). qRT–PCRs were carried out in 20 μl volume in a Qiagen RotorGeneQ High Resolution Melt Instrument (Qiagen) using a SensiFAST SYBR No-ROX One-Step Kit (Bioline, USA). Data were normalized using the mean of two housekeeping genes and the expression was calculated with 2^-ΔΔct^ formula [23]. Primers were designed using Allele ID 7 software (Premier Biosoft Intl, Palo Alto, CA, USA) according an alignment file including all of the sequences in wheat and other related plants present in the gene bank. The primers were designed according to the conserved area in those sequences in order to amplify all of the isoforms. The oligonucleotides used are listed in Table 1. For each sample, the subsequent qRT-PCR reactions were performed twice under identical conditions.

**Table 1:** List of primers.

Genes name	Orientation	Sense 5′-3′ sequence
**NR**	Forward	GGCAACTTCGTCATCAAC
	Reverse	CATCTCCGTCTCGTCCTC
**NiR**	Forward	ACACCAACCTCCTCTCCTCCTAC
	Reverse	CACAAGATAACACGGCAGCAACG
**NADH-GOGAT**	Forward	TCATCCAGCCGACCAACACG
	Reverse	CCACAATCCATACAACGAGCAGAC
**Fd-GOGAT**	Forward	GCTGATGCTGCTGTGC
	Reverse	CAAATGCTGGTGGATGGC
**GS1**	Forward	GTGGATGCCGTGGAGAAG
	Reverse	GCTGAAGGTGTTGATGTCG
**GS2**	Forward	CTCGTCCGCGTCCTTGTCCG
	Reverse	GCCGACCTGCCCCGCACG
**GAPDH**	Forward	CGAAGCCAGCAACCTATGAT
	Reverse	CAAAGTGGTCGTTCAGAGCA
**Actin**	Forward	ACCTTCAGTTGCCCAGCAAT
	Reverse	CAGAGTCGAGCACAATACCAGTTG

### NR activity

NR activity was assayed in leaves using the method described by *Li* et al. [24]. Leaf tissues were weighed and immediately ground in liquid nitrogen. Two ml of extraction buffer containing 2% (w/v) polyvinylpyrrolidone, 2 mM EDTA, 10% (v/v) glycerol and 1 mM DDT were added to 300 mg of frozen leaf powder and centrifuged at 12,000 g for 15 min. Aliquots (300 μl) of supernatants were added to 300 μl reaction medium (5 mM KNO_3_, 0.2 mM NADH) and incubated for 15 min at 25 C. The reaction was stopped by adding zinc acetate (100 μl, 0.5 M). The mixture was centrifuged at 8000 g for 5 min and the supernatant was used to determine nitrite production after the addition 300 μl of Greiss reagent (2% sulphanylamide in 5% H_2_PO_4_ and 0.1% 1-Naphthyl ethylenediamine dichloride) and reading the solution absorbance at 540 nm (Lambda 25 UV/VIS Spectrometer, Waltham, USA). The amount of NR required for the production of 1 μmol nitrite per min was defined as one unit of the NR.

### Total GS activity

300 mg wheat leaf tissues was ground in liquid nitrogen and homogenized in 2 ml extraction buffer (1 mM EDTA, 50 mM Tris buffer, 10% [v/v] glycerol, and 5 mM 2-merceptoethonal, pH 8.0). The homogenates were centrifuged at 14,000 g for 5 min and the supernatant was analyzed for total GS activity. One ml of the leaf extract were incubated with 2 ml reaction buffer (50 mM imidzole, 30 mM MgCl_2_, 25 mM hydroxylamine and 100 mM L-glutamate) at 37°C for 40 min. The reaction was terminated by adding an acidic FeCl_3_ solution, containing 80 mM FeCl_3_, 700 mM HCl, and 200 mM trichloroacetic acid. The product (formation of γ-glutamyl monohydroxamate) was measured spectrophotometrically by absorbance at 540 nm. One unit of enzyme was defined as amount of enzyme required to produce 1 μmol of glutamyl monohydroxamate per min [25].

### NO content

Nitric oxide content was determined using the method described by *Zhou* et al. [26] with slight modifications. 300 mg leaves were ground in a mortar and pestle in 2 ml 50 mM cool acetic acid buffer (pH 3.6, containing 4% zinc diacetate). After centrifugation in a refrigerated (4°C) centrifuge at 8,000 g for 15 min, homogenate was filtered. After vortexing and filtering, 500 μl of the Greiss reagent was added to 500 μl of filtrate, kept at room temperature for 30 min and the solution absorbance was read at 540 nm. Sodium nitrite was used to calculate the NO content.

### Statistical analysis

The results are shown as mean values and standard deviations of three replicates. Experimental Data was analysed as a completely randomized design (CRD) by one-way analysis of variance (ANOVA) followed by Duncan’s multiple range test (P < 5%). The statistical analyses were conducted with SPSS software ver. 16 (SPSS Inc., Chicago, IL, USA).

## Results

### SNP as NO donor increased wheat nitrogen assimilation pathway gene expression under low nitrogen supply

At low level (4 mM) of NHCl, application of NO increased most of those genes’ expression in both cultivars. However, the expression of GS1 in cv. Spitfire decreased and the expression of Fd-GOGAT and NADH-GOGAT in cv. Westonia was not affected by exogenous NO (Table 2). Growing under low KNO_3_ supply, the maximum expression of NR, NiR, NADH-GOGAT, Fd-GOGAT and GS2 of cv. Spitfire occurred in 100 μM SNP. The GS1 expression was maximum in control plants and decreased in 20 μM SNP (Table 2). Application of SNP did not affect most genes’ expression in Westonia cultivar under low KNO_3_ with the exception of GS1. The expression of GS1 showed an increase in 100 μM SNP with 4 mM KNO_3_ (Table 2). 100 μM SNP with 4 mM NH_4_NO_3_, caused an increase in the expression of NR, NiR, GS1, GS2 and Fd-GOGAT, while the expression of NADH-GOGAT was not affected by the SNP application in cv. Spitfire. When cv. Westonia was grown in 4mM NH_4_NO_3_, the SNP did not affect the expression of NiR, Fd-GOGAT and GS2 while the mRNA expression levels of NR and NADH-GOGAT decreased in 20 μM SNP. The GS1 expression increased in both 20 and 100 μM SNP under low NH_4_NO_3_ in cv. Westonia (Table 2).

**Table 2:** Effects of low nitrogen (4 mM) on mRNA expression.

N Sources	Cultivars	NO Rates	Relative Expression (%)					
			NR	NiR	NADH-GOGAT	Fd-GOGAT	GS1	GS2
**NHCl**	Spitfire	Control	1 ^c^	1 ^b^	1 ^b^	1 ^c^	1 ^a^	1 ^c^
		20 μM SNP	2.06 ^b^	2.26 ^a^	1.27 ^ab^	2.43 ^b^	0.61 ^b^	2.24 ^b^
		100 μM SNP	3.16 ^a^	2.68 ^a^	1.58 ^a^	3.09 ^a^	0.92 ^a^	2.98 ^a^
	Level of significance		0.002	0.003	0.025	0.001	0.022	0.002
	Westonia	Control	1 ^c^	1 ^b^	1 ^a^	1 ^ab^	1 ^b^	1 ^b^
		20 μM SNP	1.67 ^b^	1.73 ^a^	0.83 ^a^	0.78 ^b^	0.83 ^b^	0.88 ^b^
		100 μM SNP	2.79 ^a^	2.25 ^a^	1.01 ^a^	1.21 ^a^	1.62 ^a^	1.35 ^a^
	Level of significance		0.003	0.009	ns	ns	0.016	0.009
**KNO**_**3**_	Spitfire	Control	1 ^c^	1 ^b^	1 ^b^	1 ^a^	1 ^b^	1 ^a^
		20 μM SNP	1.89 ^b^	2.33 ^a^	1.07 ^b^	1.13 ^a^	1.27 ^a^	1.17 ^a^
		100 μM SNP	2.4 ^a^	2.8 ^a^	1.38 ^a^	1.17 ^a^	0.67 ^c^	1.21 ^a^
	Level of significance		0.004	0.006	0.026	ns	0.009	ns
	Westonia	Control	1 ^b^	1 ^a^	1 ^a^	1 ^a^	1 ^b^	1 ^a^
		20 μM SNP	1.25 ^a^	1.28 ^a^	0.97 ^a^	0.94 ^a^	0.73 ^c^	0.96 ^a^
		100 μM SNP	1.24 ^a^	1.19 ^a^	1.11 ^a^	1.02 ^a^	1.36 ^a^	1.03 ^a^
	Level of significance		ns	ns	ns	ns	0.006	ns
**NH**_**4**_**NO**_**3**_	Spitfire	Control	1 ^b^	1 ^b^	1 ^a^	1 ^b^	1 ^c^	1 ^b^
		20 μM SNP	1.29 ^b^	2.24 ^b^	0.97 ^a^	0.9 ^b^	2.92 ^b^	1.04 ^b^
		100 μM SNP	2.85 ^a^	4.57 ^a^	0.96 ^a^	1.71 ^a^	3.97 ^a^	1.41 ^a^
	Level of significance		0.002	0.004	ns	0.007	0.001	0.028
	Westonia	Control	1 ^a^	1 ^a^	1 ^a^	1 ^a^	1 ^b^	1 ^a^
		20 μM SNP	0.66 ^b^	0.83 ^a^	0.71 ^b^	0.81 ^a^	1.61 ^a^	0.78 ^a^
		100 μM SNP	1.01 ^a^	1.01 ^a^	1.24 ^a^	1.06 ^a^	1.77 ^a^	1.04 ^a^
	Level of significance		0.041	ns	0.015	ns	0.025	ns

Different letters meaning significantly different at 5% levels as calculated by Duncan multiple test.

### SNP supplied with high nitrogen concentration increased nitrogen assimilation expression in Spitfire but not in Westonia

In NHCl treated plants, NO did not affect the expression of NR, GS1, Fd-GOGAT and NADH-GOGAT, while NiR showed a decreased expression in the presence of 100 μM SNP in cv. Spitfire. In cv. Westonia, NO caused an increase in the expression of GS1 and a decrease of the NADH-GOGAT expression while the expression of the other genes was not affected by NO (Table 3). Application of SNP in KNO_3_ treated plants did not affect the expression levels of GS2, Fd-GOGAT and NADH-GOGAT in cv. Spitfire; however it increased the expression of NiR and GS1 and decreased the expression of NR. In cv. Westonia SNP application caused a decrease in the expression of NR and GS2 while the expression of other genes was not affected (Table 3). When treated with NH_4_NO_3_, SNP plus 40 mM nitrogen treatment in cv. Spitfire caused an increase in the expression of NR, NiR and Fd-GOGAT genes but did not affect the NADH-GOGAT, GS1 and GS2 expression. Application of SNP in cv. Westonia with 40 mM NH_4_NO_3_ caused a decreased expression of NR, NiR and NADH-GOGAT while the expression of Fd-GOGAT, GS1 and GS2 was not affected (Table 3).

**Table 3:** Effects of high nitrogen (40 mM) on mRNA expression.

N Sources	Cultivars	NO Rates	Relative Expression (%)					
			NR	NiR	NADH-GOGAT	F-GOGAT	GS1	GS2
**NHCl**	Spitfire	Control	1 ^a^	1 ^a^	1 ^a^	1 ^a^	1 ^b^	1 ^b^
		20 μM SNP	0.9 ^a^	0.79 ^ab^	1.12 ^a^	1.089 ^a^	1.28 ^a^	1.95 ^a^
		100 μM SNP	0.77 ^a^	0.63 ^b^	1.01 ^a^	1.01 ^a^	1.23 ^ab^	1.29 ^b^
	Level of significance		ns	0.043	ns	ns	ns	0.012
	Westonia	Control	1 ^a^	1 ^a^	1 ^a^	1 ^a^	1 ^c^	1 ^a^
		20 μM SNP	0.71 ^a^	0.82 ^a^	0.62 ^b^	0.73 ^a^	1.47 ^b^	0.74 ^a^
		100 μM SNP	0.96 ^a^	0.89 ^a^	0.77 ^b^	0.94 ^a^	2.05 ^a^	0.98 ^a^
	Level of significance		ns	ns	0.022	ns	0.004	ns
**KNO**_**3**_	Spitfire	Control	1 ^a^	1 ^b^	1 ^a^	1 ^a^	1 ^b^	1 ^a^
		20 μM SNP	1.05 ^a^	1.43 ^a^	1.09 ^a^	1.12 ^a^	1.42 ^a^	1.13 ^a^
		100 μM SNP	0.61 ^b^	0.84 ^b^	1.04 ^a^	0.91 ^a^	0.94 ^b^	0.93 ^a^
	Level of significance		0.016	0.008	ns	ns	0.021	ns
	Westonia	Control	1 ^a^	1 ^a^	1 ^a^	1 ^a^	1 ^a^	1 ^a^
		20 μM SNP	0.87 ^ab^	0.9 ^a^	0.99 ^ab^	0.91 ^ab^	0.7 ^a^	0.76 ^ab^
		100 μM SNP	0.78 ^b^	0.89 ^a^	0.78 ^b^	0.66 ^b^	0.82 ^a^	0.6 ^b^
	Level of significance		0.043	ns	ns	ns	ns	0.03
**NH**_**4**_**NO**_**3**_	Spitfire	Control	1 ^c^	1 ^b^	1 ^b^	1 ^b^	1 ^b^	1 ^a^
		20 μM SNP	1.42 ^b^	1.55 ^a^	1.17 ^ab^	1.14 ^b^	1.39 ^a^	0.96 ^a^
		100 μM SNP	1.85 ^a^	1.56 ^a^	1.46 ^a^	1.7 ^a^	1.35 ^a^	1.18 ^a^
	Level of significance		0.01	0.019	ns	0.014	ns	ns
	Westonia	Control	1 ^a^	1 ^a^	1 ^a^	1 ^a^	1 ^a^	1 ^a^
		20 μM SNP	0.72 ^b^	0.69 ^b^	0.65 ^b^	0.93 ^a^	0.7 ^b^	0.99 ^a^
		100 μM SNP	0.47 ^c^	0.54 ^b^	0.59 ^b^	0.84 ^a^	0.98 ^a^	0.96 ^a^
	Level of significance		0.001	0.03	0.008	ns	ns	ns

Different letters meaning significantly different at 5% levels as calculated by Duncan multiple test.

### Role of NO in NR activity

**Fig 1:**
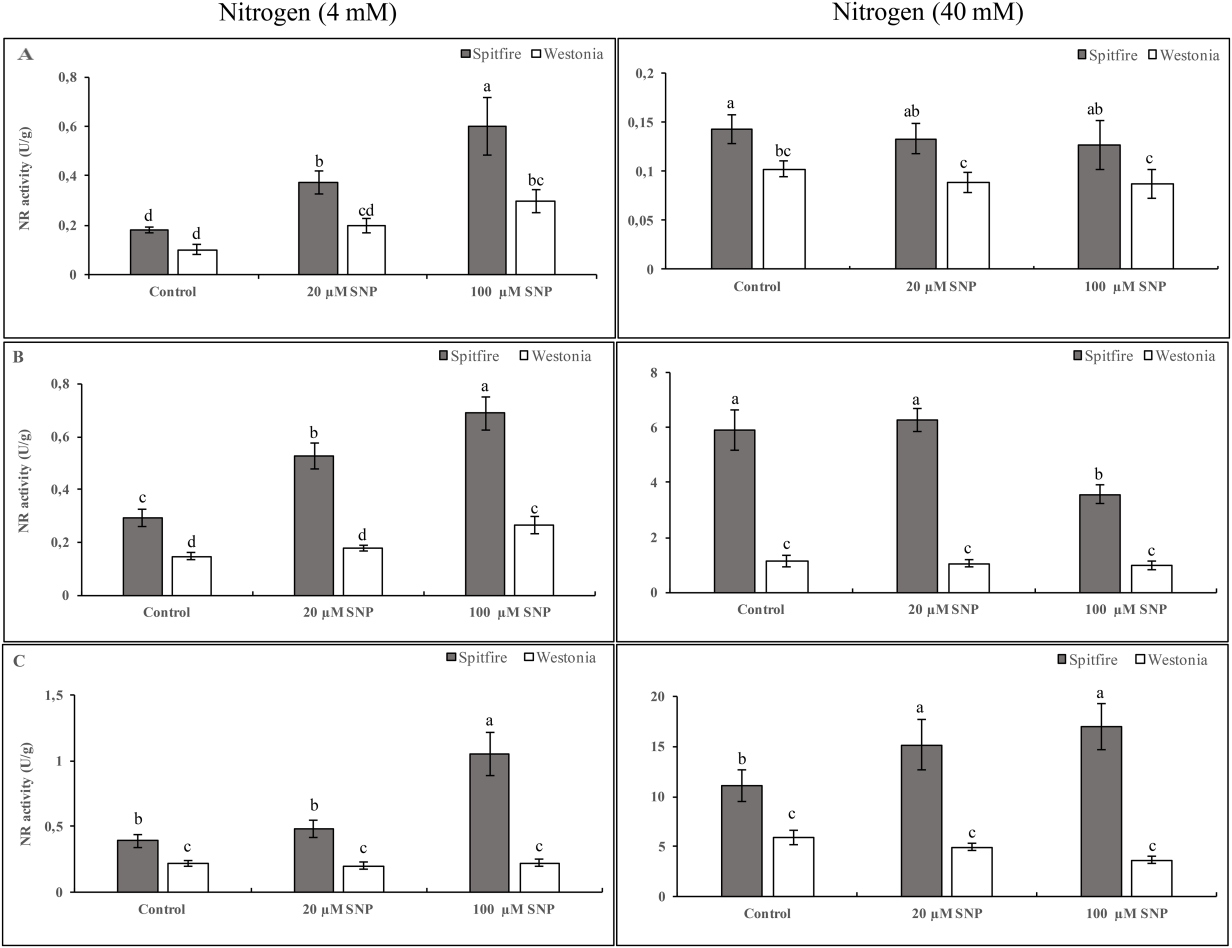
Effects of NO and nitrogen sources on NR activity. Wheat seedlings (cvs Spitfire and Westonia) were grown in glasshouse for two weeks. Then, plants were irrigated with nitrogen-free nutrient solution for a week. Plants treated with different concentrations (left = 4mM and right = 40 mM) and different chemical forms of nitrogen (A = NHCl, B = KNO_3_ and C = NH_4_NO_3_) with 0 (as a control), 20 or 100 μM of SNP for 3 days. Leaf tissues were harvested 24 hours after nitrogen treatments. NR activity was measured on three biological repeats. Different letters mean significantly different at 5% levels as calculated by Duncan multiple test.

The activity of NR increased by SNP application in both cultivars treated with 4 mM NHCl. However, when the NHCl level increased to 40 mM, SNP did not affect NR activity (Fig 1A). Both cultivars showed an increase in the activity of NR in 4 mM KNO_3_ plus SNP. However, at 40 mM KNO_3,_ SNP did not affect NR activity in cv. Westonia while it decreased NR activity in cv. Spitfire (Fig 1B). In NH_4_NO_3,_ the two cultivars showed different responses to SNP application. In both low and high nitrogen supplies, SNP increased the activity of NR in cv. Spitfire but not in cv. Westonia (Fig 1C). In all three nitrogen sources the activity of NR in cv. Spitfire was significantly higher than the activity of NR in cv. Westonia (Fig 1).

### Effects of NO on total GS activity

**Fig 2:**
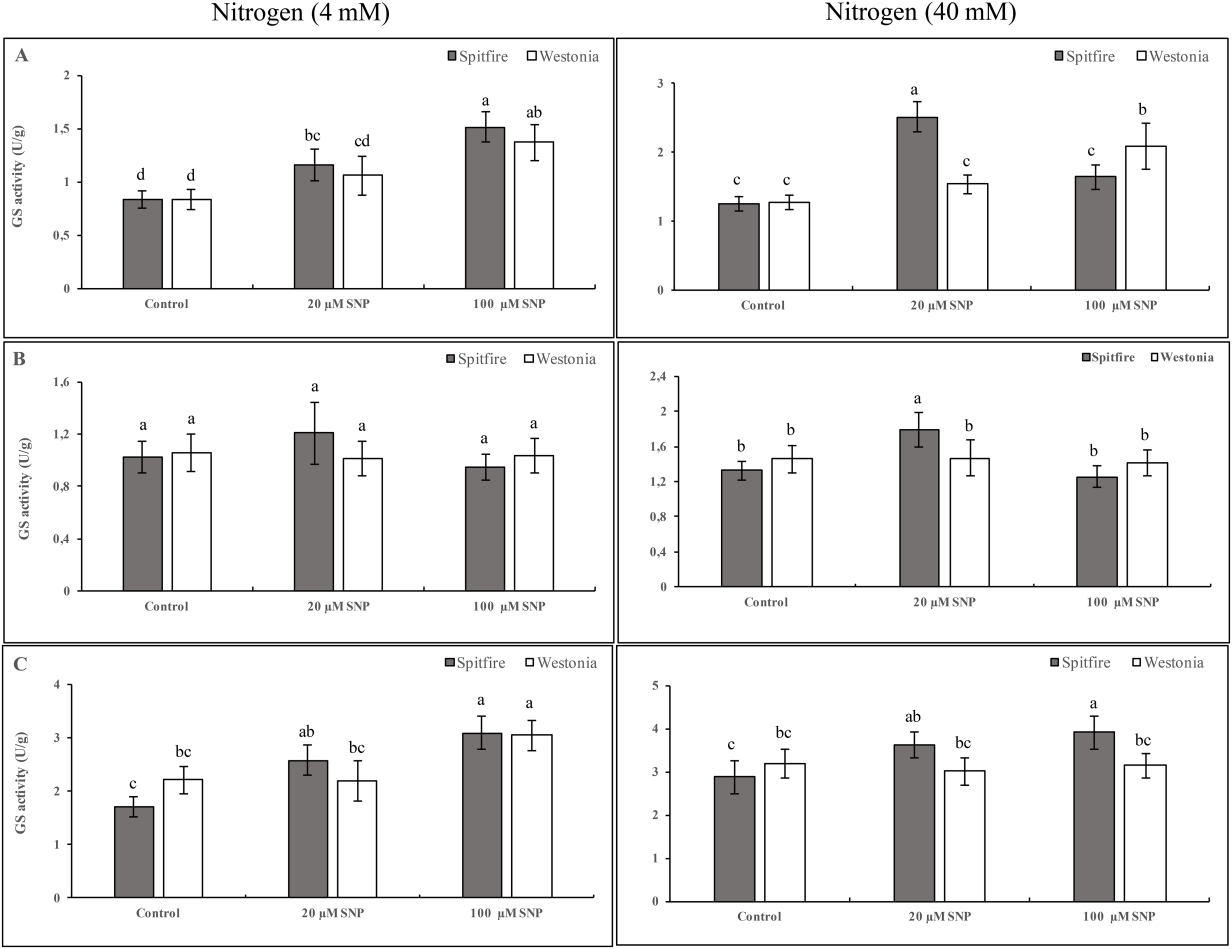
Effects of NO and nitrogen sources on GS activity. Wheat seedlings (cvs Spitfire and Westonia) were grown in glasshouse for two weeks. Then, plants were irrigated with nitrogen-free nutrient solution for a week. Plants treated with different concentrations (left = 4mM and right = 40 mM) and different chemical forms of nitrogen (A = NHCl, B = KNO_3_ and C = NH_4_NO_3_) with 0 (as a control), 20 or 100 μM of SNP for 3 days. Leaf tissues were harvested 24 hours after nitrogen treatments. GS activity was measured on three biological repeats. Different letters meaning significantly different at 5% levels as calculated by Duncan multiple test.

At the 4 mM NHCl level, 100 μM SNP showed maximum activity of GS in their leaves. At the 40 mM NHCl level, cv Spitfire showed the highest GS activity with 20 μM SNP. Overall, 100 μM SNP was the best treatment for maximum GS activity of cv. Westonia in plants growing in 4 mM and 40 mM NHCl (Fig 2A). The activity of GS was not affected by SNP application in low and high KNO_3_ treatments in cv. Westonia while in cv. Spitfire 20 μM SNP caused a significant increase in GS activity in 40 mM KNO_3_ treatments (Fig 2B). In plants treated with NH_4_NO_3,_ SNP application increased the activity of GS in low and high nitrogen treatments in cv. Spitfire. In low NH_4_NO_3_, 100 μM SNP caused a significant increase in the activity of GS in cv. Westonia while in 40 mM NH_4_NO_3,_ SNP application did not affect its GS activity (Fig 2C).

### Effects of nitrogen forms and SNP on NO accumulation in wheat leaves

**Fig 3:**
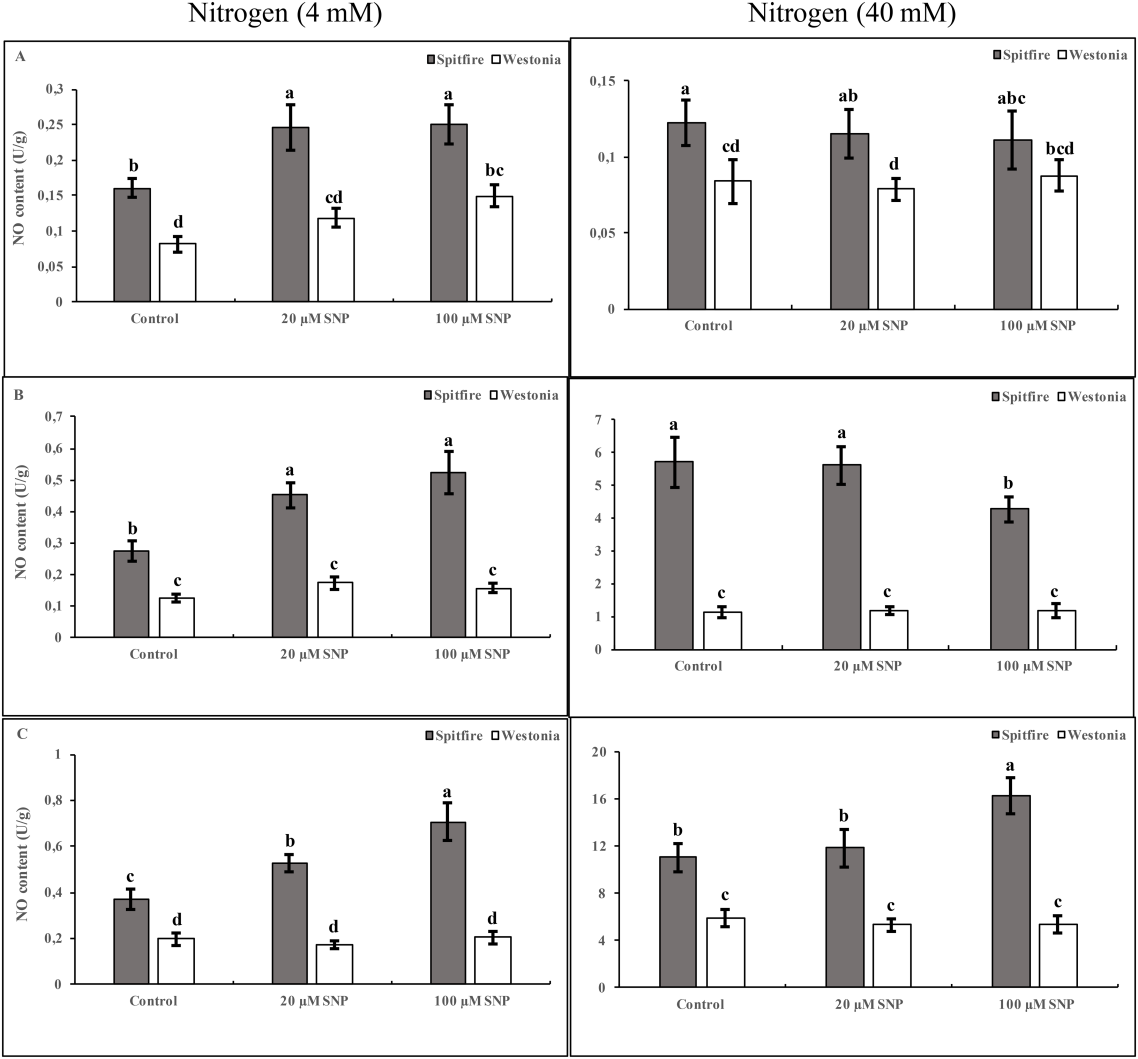
Effects of NO and nitrogen sources on NO content. Wheat seedlings (cvs Spitfire and Westonia) were grown in glasshouse for two weeks. Then, plants were irrigated with nitrogen-free nutrient solution for a week. Plants treated with different concentrations (left = 4mM and right = 40 mM) and different chemical forms of nitrogen (A = NHCl, B = KNO_3_ and C = NH_4_NO_3_) with 0 (as a control), 20 or 100 μM of SNP for 3 days. Leaf tissues were harvested 24 hours after nitrogen treatments. NO content was measured on three biological repeats. Different letters meaning significantly different at 5% levels as calculated by Duncan multiple test.

In plants treated with 4 mM NHCl, SNP treatment increased the NO accumulation in both cultivars as compared with control. However, in the high NHCl treated plants, SNP did not affect the internal NO in both cultivars (Fig 3A). Maximum NO accumulation in cv. Spitfire growing in low level of KNO_3_ was observed when treated with 20 and 100 μM SNP while in high KNO_3_ treatments SNP application caused a decrease in the activity of GS. In cv. Westonia NO did not affect GS activity in both low and high KNO_3_ treated plants (Fig 3B). In plants treated with NH_4_NO_3_, the application of SNP increased NO accumulation in both low and high nitrogen levels in cv. Spitfire but not in cv. Westonia (Fig 3C).

## Discussion

Previous studies have indicated that the expression levels of different key genes for nitrogen metabolism are affected by different nitrogen sources such as NO_3_^−^ or NH_4_^+^ [22, 27, 28]. In addition, it has been shown that the nitric oxide generated by nitrate reductase influences the plant nitrogen metabolism pathway [19, 29, 30]. In the present study, in order to explore the potential application of these findings, we examined the expression and the activity of nitrogen metabolism key enzymes in response to different combinations of nitrogen and sodium nitroprusside. The cv. Spitfire is known as good yielding cultivar with large grain size and high protein content. In our study we also used cv. Westonia, as a reference, which is a common wheat cultivar in Australia with average performance including yield and most other traits.

Nitrate reductase is the most important enzyme in NO_3_^-^ assimilation pathway in plants and its expression and activity increase by nitrate application while NH_4_^+^ as the end product in the nitrate reduction pathway reduces its activity and expression [31]. Results presented here indicate that SNP application increases the expression/activity of NR in wheat seedlings under low NHCl treatments. These results show that NO treatment alleviates NH_4_^+^ toxicity in wheat seedlings under low nitrogen treatments (Table 2). This protective effect of NO against NH_4_^+^ toxicity may be related to its ability to react with some ROS produced under these conditions, making NO act as a chain breaker and showing its proposed antioxidant properties [32]. Moreover, it has been reported that NO can react with lipid alkoxyl (LO˙) and peroxyl (LOO˙) radicals, leading to the conclusion that NO could stop the propagation of radical-mediated lipid oxidation in a direct fashion [33]. Thus NO may help plants to survive under stressful conditions through its action as signaling molecule to activate antioxidant enzymes and reacting with active oxygen and lipid radicals directly. However, it has also been proved that NO affects plant metabolism in a concentration-dependent pattern [34, 35].

Also recent studies have shown that NO generated by the NR pathway plays a pivotal role in improving the N acquisition capacity by increasing both lateral root (LR) initiation and the inorganic N uptake rates [19]. Therefore, it is possible that NO causes changes in both root structure and nitrate transporter (NRT) in root cells resulting in an increase in NO_3_^-^ uptake from the soil [36–38]. Nitrate acts as a signal to trigger a number of molecular and physiological events that lead to the overall response of a plant to N availability [39]. NR is known as an inducible enzyme and its expression corresponds with the increase in the levels of NO_3_^-^ present. In our study, the application of SNP in plants growing under low N supplies might increase the plants internal N content and causing an increase in the expression/activity of the nitrogen assimilation pathway enzymes.

Regulation of NR and GS may occur at the levels of transcription, translation, subcellular localization, subunit assembly and post-translational modification of the protein and its turnover [40, 41]. It has been found that NR activity is inhibited by NO in *Triticum aestivum* [42] and also in *Chlamydomonas reinhardtii* [18]. Our results clearly showed that the effects of NO on the activities of NR and GS enzymes depend on available N forms and concentrations, NO concentrations, and wheat genotypes (Figs 1 and 2). As mentioned above, at low N availability, NO causes an increase in internal nitrogen by affecting root ramification and possibly nitrogen transporters. At high concentration of N supplied, NO could inhibit the triggering of phosphorylation and 14-3-3 binding and/or being involved in an alternative regulation of the enzyme with the formation of S-nitrosothiols (SNO) as a result of NO addition to cysteine thiols [43]. It has been shown that in tobacco plants that the N-terminal region of NR is involved in the complete inhibition during light/dark transition independent of 14-3-3 proteins and their phosphorylation [44].

## Conclusion

In summary, our data show that the signaling molecule NO may have an important role in transcriptional and post-transcriptional regulation of nitrogen assimilation pathway enzymes (please see supplementary data S1 to compare mRNA expression, enzyme activity and also NO content). While its inducible effects on assimilation enzymes at low N concentrations can be observed, at high N concentrations such inductions are absent. This may represent a strategy by which wheat plants adopt to increase their nitrogen use efficiency.

## Supporting information

S1 Dataset Raw dataset providing all original data for the experiment. (XLSX)
